# Quality Attributes of Sesame Butter (Tahini) Fortified with Lyophilized Powder of Edible Mushroom (*Agaricus blazei*)

**DOI:** 10.3390/foods11223691

**Published:** 2022-11-17

**Authors:** Hatem Salama Ali, Ahmed Noah Badr, Tawfiq Alsulami, Mohamed Gamal Shehata, Mohamed Mahmoud Youssef

**Affiliations:** 1 Food Technology Department, National Research Center, Dokki, Cairo 12622, Egypt; 2 Food Toxicology and Contaminants Department, National Research Centre, Dokki, Cairo 12622, Egypt; 3 Food Science and Nutrition Department, Food and Agriculture Science College, King Saud University, Riyadh 12372, Saudi Arabia; 4 Food Science Department, Arid Lands Cultivation Research Institute, City of Scientific Research and Technological Applications (SRTA-City), New Borg El-Arab 21934, Egypt; 5 Food Research Section, R&D Division, Abu Dhabi Agriculture and Food Safety Authority (ADAFSA), Abu Dhabi P.O. Box 52150, United Arab Emirates; 6 Food Science and Technology Department, Faculty of Agriculture, Alexandria University, Alexandria 21545, Egypt

**Keywords:** *Agaricus blazei*, aflatoxins, tahini, quality attributes, chemical composition, emulsion stability, oxidative stability, zearalenone

## Abstract

Sesame butter (tahini) is a common appetizer and food additive in the Mediterranean basin. Pathogenic strains and mycotoxin content are the most hazardous issues in the final product. This investigation aimed to enhance the quality and safety properties of tahini products against microbial hazards and mycotoxins. Local samples of tahini were evaluated for natural contamination, including mycotoxin level determinations. *Agaricus blazei* was utilized as a bioactive source and evaluated for the bioactive content of laccase, *B-glucan*, antioxidant activity, and phenolic content, as well as antimicrobial and antioxidant potency. Two fortification ratios (0.5% and 1.0%) were chosen to apply *Agaricus* in tahini sesame as a model. Chemical composition, color attributes, sensory properties, emulsion, and oxidative stability were evaluated for the fortified samples versus the control. The results reflected increments of protein (22.91 ± 0.64% to 29.34 ± 0.96%), fiber content (3.09 ± 0.05% to 6.27 ± 0.06%), emulsion stability (84.9 ± 1.24% to 95.41 ± 0.56%), oxidative stability, and bioactive group content. The fortification process is reflected by the absence of *Salmonella*, *Listeria*, and *E. coli* bacteria from contaminated samples after 30 days of storage. The water activity for 1.0% fortification (0.154 ± 0.001) was recorded as lower than the control sample (0.192 ± 0.002). Moreover, the degradation of aflatoxins and zearalenone content was recorded during storage. The degradation ratio reached 68% and 97.2% for 0.5% and 1.0% fortifications, respectively, while zearalenone degradation recorded a decline of 26.7% and 33.7%, respectively, for the same fortification ratios. These results recommended 1.0% lyophilized mushroom fortification as a quality and ameliorative safety treatment for tahini products.

## 1. Introduction

Tahini is an appetizer that precedes the main dish in restaurant meals. It can be served crudely with little vinegar drops or as a salad by mixing it with yogurt [1]. Tahini has nutritional and biological benefits since it contains lipids, proteins, dietary fiber, niacin, thiamin, and minerals [2]. Tahini is a popular food material consumed in the Mediterranean basin and Middle East countries [3,4]. It is also known in North America and Canada due to its utilization in restaurant dishes [5]. Tahini may also be mixed with molasses in a traditional container, particularly consumed and preferred by children [6]. Tahini can be applied to popular dishes such as fava beans (*Foul*), consumed in Egypt as a staple food [7]. This kind of food generally uses bulk tahini packed in large containers. Tahini is mainly prepared from hulled seeds of white sesame, while some varieties may contain peanuts or chickpeas [8], according to the local culture of the manufacturing place. Tahini looks like a puree or paste in texture and has a gray-yellow color.

Recently, food safety and security have been basic human needs linked with several issues and public health problems [9]. Microbial contamination is related to food materials, processing, handling, and storage. Tahini mainly consists of sesame paste and is exposed to pathogens and mycotoxin contaminations [10]. This contamination may occur due to the raw material or the processing and handling steps. Tahini is linked to recent foodborne illnesses, and its product was recalled due to its microbial contamination by *Salmonella*, *Listeria monocytogenes*, and coliforms, including *E. coli* O157:H7 [10,11]. Aflatoxin-producing fungi and their linked toxins threaten sesame seeds before and after seed harvesting [12]. Sesame and peanut are highly contaminated by toxigenic fungi, particularly those that produce aflatoxins (AFs) as secondary metabolites [13]. AFs are exceedingly harmful natural toxic compounds created essentially by fungal organisms drawn to oilseeds, cereals, and nuts, where health and economic difficulties could have occurred.

The results of Sabry et al. [14] on Egyptian varieties of sesame showed they were typically infected with various fungi, some of which are mycotoxigenic and can harm human health and the economy. That investigation characterized the fungal infection linked to Egyptian sesame seeds, particularly aflatoxigenic strains. Isolated fungi belong to different genera, including *Aspergillus* as the dominant fungi (up to 91.66%). Meanwhile, *A. flavus* and *A. parasiticus* were the most isolated genus, followed by *A. niger.* These results pointed out the significance of sesame seed treatment before food application. Antibacterial and antifungal components could be a suitable solution against toxigenic fungi contamination and their related metabolites [15,16].

AFs are secondary metabolites produced by *Aspergillus* fungi that naturally contaminate foodstuffs, causing toxic impacts on vertebrates [17]. This contamination can happen on the field to the fork during production. Humans are typically exposed to mycotoxins by consuming contaminated food [18]. Various studies have shown a significant frequency of aflatoxin exposure globally in oilseed crops and their based products, including peanuts and sesame [14,19]. AFs are categorized as predominantly affecting the liver, and the International Agency of Research on Cancer (IARC) recognized them as Class 1 carcinogen compounds [20]. They also exert a toxic impact on the different biological systems inside the body.

Since ancient times, humans have revered mushrooms as a culinary marvel, used in fine dining establishments worldwide for their distinctive taste. In addition to this, they are well acclaimed for the health benefits they provide when consumed. Proteins, polysaccharides, steroids, vitamins, nucleotides, enzymes, flavonoids, glycoproteins, amino acids, and polyphenols are only some of the many phytochemicals found in mushrooms. Mushrooms have improved immunity thanks to their high protein, minerals, vitamins, fiber, and low-fat concentrations [21]. *Agaricus blazei* is a traditional mushroom that has been used for centuries for its purported health benefits, including its ability to alleviate symptoms of anxiety and depression, boost the immune system, treat diabetes, lower cholesterol, ward off bone loss and stomach ulcers, ease digestive issues, and even combat cancer [22]. The mushroom’s medical use in traditional Eastern medicine has been well documented and explored as a new functional food during the last decade. Mushroom extracts that dissolve in hot water have a long medical tradition in East Asia (Firenzuoli).

Mushrooms are an excellent food choice since they include several essential nutrients, such as vitamins and unsaturated fatty acids (particularly vitamins B and C). With their high concentrations of bioactive ingredients, mushrooms have a potent antioxidant effect and can thus help ward off conditions such as cancer, diabetes, and heart disease. Mushrooms might be a source of compounds with antimicrobial and antifungal effects. To prevent oxidative damage, the body needs mycochemicals, which are molecules that neutralize free radicals [23]. Mycotoxins and other free radicals (chemicals with one or more unpaired electrons) cause biological damage and play a role in many chronic diseases [24].

In this regard, contamination in tahini products varies between pathogenic bacteria and mycotoxins. Previous studies have tried to avoid bacterial contamination in tahini using microwave heating [11], while mycotoxin risk still exists. The present study aims to prevent tahini contamination during manufacturing and handling steps up to the final consumer. Providing the safety and quality properties of tahini as a common product was the main target of the present study. Utilizing bioactive fortification by medicinal mushrooms is a neoteric application for the critical function of contamination avoidance. Tahini types, which are locally used, were investigated for natural contamination. In contrast, a common type (tahini of crude sesame) was utilized as a model to evaluate the fortification impact regarding the nutritional and microbial benefits.

## 2. Materials and Methods

### 2.1. Sample Collection

By surveying the bulk tahini that is handled in markets, there were 3 types known as the dominant products in descending order: sesame tahini (STn), peanut tahini (PTn), and chickpea tahini (CTn). Random samples (20 bottles/500 g) of each type were collected for further evaluation. The mushroom plant of *Agaricus blazei* was purchased from a local herbal market and lyophilized using a lyophilizer tray system (Laboratory Lyophilizer, FD-10-MR Multi-Manifold, Esquire Biotech, Chennai, India). The dried slices were ground to a fine powder, passed through a 20-mesh sieve (850 microns), and stored in a graduated bottle (500 mL, with a seal cap).

### 2.2. Microbial Evaluation

Collected samples of STn, PTn, and CTn were evaluated for total count according to the methodology described by Smith and Townsend [25]. Total count media (HiMedia Laboratories Pvt. Limited, Mumbi, India) were prepared, autoclaved (121 °C/15 min), and poured into Petri dishes (15 mL). In Brief, each sample’s serial dilutions (using peptone buffer) were performed to achieve a colony count of fewer than 300 colonies/plate. The medium was inoculated by pour plating and spread plating and incubated (at 28 °C for 72 h). Selective media were applied for each strain to investigate the presence of pathogenic bacterial strains of *Staphylococcus*, *Salmonella*, *Listeria*, and *E. coli*.

### 2.3. Determination of Active Components in Mushroom

The FTIR spectra were determined using the same methodology and apparatus described previously [26]. The mushroom content of the laccase was purified and determined according to the method referred to by You-Sung et al. [27]. The enzyme activity was determined using the spectrophotometric method in the presence of the ABTS+ radical (1 mM ABTS solution in pH 5.5 and 8 mM MES buffer) [28]. Briefly, laccase activity was determined in a mushroom powder supernatant (after soaking and centrifugation) by measuring the 420 nm oxidation of 2,2′-azino-bis 3-ethyl-thiazoline-6-sulfonate (ABTS+). The laccase activity after the ultrafiltration was also evaluated using syringaldazine and 2, 6 dimethoxy-phenol (DMP) as substrates. The enzyme activities were determined in a 100 mM citrate/phosphate buffer (pH 4.5) and were expressed in international units (1 unit of activity = 1 mol/min).

The percentage of β-glucan was detected in lyophilized mushroom powder after the procedure described in Khojah et al. [26]. β-glucan was measured using the mixed-linkage glucan kit (MegaZyme, NEOGEN, Lansing, MI, USA). The absorbance was measured using a Shimadzu spectrophotometer (1900i UV-Vis Spectrophotometer, Kyoto, Japan) at 510 nm wavelength.

The phenol–sulfuric acid method determined the bioactive molecules related to the carbohydrate content [29]. Briefly, powdered material was submerged in water (1:25) in a clean bottle and refluxed in a Syncore parallel reactor (Büchi, Switzerland; 100 °C, 200 rpm, 1 h). The extract was centrifuged (4500× *g*/10 min) using an Avanti J-15R centrifuge (Beckman Coulter, Fullerton, CA, USA). The supernatant was evaporated to dryness under vacuum, and then residues were redissolved in 5 mL water, followed by adding 20 mL of ethanol for precipitation of crude polysaccharides. After the solution stood (8 h/4 °C), it was centrifuged (4000× *g*/10 min). The residue was dried using the water bath (60 °C) and redissolved in hot water (4 mL). The low-molecular-weight compounds were removed thrice by centrifugation (3000× *g*/25 °C/30 min). The supernatant was transferred to an ultracentrifugal filter (molecular weight cut-off: 3 kDa; Millipore, Billerica, MA, USA), and the crude polysaccharides were then analyzed as glucose content. The concentration was calculated using a standard curve for several glucose-standard concentrations.

### 2.4. Mycotoxin Extraction and Determination

Collected samples of tahini were evaluated for their mycotoxin contents of aflatoxins (AFB1, AFB2, AFG1, and AFG2) and zearalenone (ZEA). In addition, samples after fortification with mushroom powder (0.5% and 1.0% *w*/*w*) were estimated to evaluate the changes in the fortification process. The AFs were extracted from the samples using the same methodology described by Ertekin et al. [30]. Briefly, exactly one 50 g sample was intermixed with sodium chloride (5 g) and methanol–water (100 mL, 80%) in a blender at high speed (1 min), then filtered (Whatman No. 1). The collected solution was passed through an AflaTest^®^ immunoaffinity column (VICAM, Watertown, MA, USA), where the rate was adjusted using a peristaltic pump (Golander BT100S Pump, Dechant-Heimbach-Str., Bonn 53177, Germany) connected to the chromatographic manifold unit (SPE Vacuum Manifold Chromabond^®^, Roth Carl, Karlsruhe, Germany) to collect the filtrate at a rate of 1 drops/s. Distilled water was then applied to clean the column (2 times) for the cleaning step. After the cleaning step, aflatoxin was eluted from the column with methanol (1 drop/s) into clean vails and mixed with a 1 mL AflaTest^®^ developer. Higher contaminated samples were diluted and repeated for quantification.

The same methodology extracted zearalenone except the elution was acetonitrile–water (90%), and the Zearatest^®^ column was used for the cleanup and toxin recovery. The quantitative analyses were performed using a precalibrated fluorometer (VICAM Series 4EX Fluorometer, Watertown, MA, USA; LOD, 1.0 μg/L).

### 2.5. Determination of Antibacterial and Antifungal Activities

The mushroom powder was extracted using warm diffusion extraction [31]. The extracted solution was lyophilized, and serial concentrations of dried extract were tested against bacterial and fungal strains (10, 25, and 50 µg/well). The antibacterial assay used six strains of bacteria, including *Staphylococcus aureus* ATCC 33591, *Bacillus cereus* ATCC 11778, *Enterococcus faecalis* ATCC 51299, *Salmonella typhi* ATCC 14028, *Escherichia coli* ATCC 11229, and *Listeria monocytogenes* ATCC 7644, where the methodologies followed the technique of the well-diffusion assay described by Abdel-Fatah et al. [32]. The antifungal assays were performed against *Aspergillus flavus* ITEM 698, *Penicillium chrysogenum* ATCC 10106, *Fusarium culmorum* KF 846, *A. niger* ITEM 9568, and *A. ochraceus* ITEM 7043 fungal strains. A larger inhibition zone diameter (mm) reflects the efficiency of the applied concentration. The applied incubation temperature was 37 °C/24 h for the antibacterial investigation and 25 °C/4 days for the antifungal analysis.

### 2.6. Determination of Antioxidant Activity and Phenolic Content of Agaricus blazei

The total phenolic contents of samples were estimated using Folin–Ciocalteu reagent and represented in milligrams of gallic acid equivalent per gram (mg GAE/g) compared to a blank following the methodology described in Badr et al. [33]. A UV-spectrophotometer (Shimadzu, Kyoto, Japan) detected absorbance at 760 nm.

Antioxidant activity was measured using three different assays (BPPH, ABTS, and reducing power) [34]. Briefly, the 2,2-diphenyl-1-picrylhydrazyl (DPPH) reduction was measured at 517 nm and expressed in the % DPPH reduction ratio. The 2,2′-azino-bis(3-ethylbenzothiazoline-6-sulfonic acid) diammonium salt radical cation (ABTS+) was measured at 734 nm/25 °C and represented as % ABTS scavenging activity. The absorbance was measured using a Shimadzu spectrophotometer for the reducing power evaluation [35], where ascorbic acid was utilized as a positive control, and deionized water functioned as a blank.

### 2.7. Fortification of Tahini Using Agaricus blazei

*Agaricus blazei*, as a source of bioactive components and therapy, may be capable of producing functional foods. The authors plan to apply a fortification step for only sesame tahini processing using two percentile ratios of mushroom additive (0.5% and 1.0% *w*/*w*). The sesame tahini was chosen as it is the common variety used in the commercial grade. The mushroom powder, prepared before, was used to add the requested ratios. After fortification, the product characteristics were then studied to evaluate the additional benefits that may be achieved for tahini. The resultant products of fortified tahini were homogenized using a mechanical stirrer homogenizer to ensure the best distribution, followed by a vacuuming step using a vacuum pump to avoid forming bubbles (that affect the viscosity estimation).

### 2.8. Evaluation of the Tahini Properties

The chemical composition of tahini sesame was estimated before and after the fortification step. The tahini’s carbohydrate, protein, fat, moisture, and ash contents were determined using AOAC procedures [36]. The analyses were carried out in triplicates, and the mean value per constituent was estimated. In the same way, the fiber content of tahini samples was also determined according to the methodology described by van Soest et al. [37].

The color of the control and treated samples of tahini were measured. The values of (L*, a*, and b*) were figured out using a spectrocolorimeter and the CIF lab color scale (Hunter, LabScan XE, Brandenburg, Germany). The L* scale ranges from darkness (zero) to whiteness (100); the scale ranges from positive red Chroma to negative green Chroma, and the b* scale ranges from positive yellow to negative blue [26]. The water activity of evaluated tahini samples was determined using the water activity meter of AquaLab 4TE, Pullman, WA, USA.

The tahini samples’ viscosity (centipoise) was measured at 15 °C and 37 °C, as the ambient temperature of summer and winter, respectively, using a Brookfield rotational viscometer (Model Lab-Line Instruments, Inc., Melrose Park, IL, USA) with Spindle No. 5 and an application speed of 0.5–100 rpm. A thermostatic water bath was used to keep the processing temperature constant at (±1 °C), and adequately sized samples (300 mL) were used for viscosity measurements. Product shelf-standing estimated the emission stability (EE) of tahini products (fortified and nonfortified) for 60 days of storage. The separated oil amounts were measured every 15 days, and the EE was calculated as follows:

$$\%\;EE=\frac{\mathrm{Tv}-\mathrm{Ov}}{\mathrm{Tv}}\;\times \;100$$

where Tv: total tahini amount used for the test; Ov: oil content separated during shelf storage.

### 2.9. Determination of Oxidative Stability

The peroxide value (PV) and *p*-anisidine value (AV) were determined in the separated amount of oil collected from the bottle’s surface used for storing tahini samples according to the method described by Gordon et al. [38]. The *p*-anisidine value was measured at 350 nm for 1 g of oil, mixed with 100 mL of a mixture of solvent and reagent (isooctane solution with 0.25% *p*-anisidine in glacial acetic acid). The PV represents primary oxidation products (peroxides and hydro-peroxides), while *p*-anisidine represents secondary oxidation products (aldehydes and ketones).

### 2.10. Determination of the Changes in Microbial Content

Due to the experimental fortification design, where the mushroom powder was applied in crude sesame tahini as a model, the changes in microbial load of tahini type were estimated at fortification ratios compared to the control. The methodology utilized in this experiment was mentioned in Section 2.2.

### 2.11. Sensory Evaluation of Tahini

The sensory evaluation of tahini samples was performed in conformity with a standard test (UNI, 10957:2003) [38], employing 40 panelists ranging in age from 20 to 55, consisting of 15 males and 25 females for the panel test. The panelist assessment was held at the Food Industries and Nutrition Institute of the National Research Centre (NRC). Tahini samples were presented in a randomized order. Panelists were asked to record their evaluation of color, taste, odor, texture, consistency, and general acceptability. Their impressions were rated on an 8-point hedonic scale (1 = poor tahini product, 4 = acceptable tahini product, 8 = preferable tahini).

### 2.12. Statistical Evaluation

All tests were carried out in triplicate, and the results were presented as means of standard deviation. The data were analyzed using Statistical Software for the Social Sciences (SPSS V.16). The analysis of variance (ANOVA) was performed to determine if there was a significant difference between the mean values, and Duncan’s multiple range test (*p* = 0.05) was computed.

## 3. Results

### 3.1. Natural Contamination of Bulk Tahini Samples

Popularly, tahini is made in three types of paste, with sesame as the main type and sometimes peanut or chickpea paste. As a starting point of the present research, these types were evaluated for their level of contamination under local conditions. The survey of common tahini samples handled in markets reflects a considerable contamination degree by pathogenic bacterial strains, with a high content of total count bacteria (Table 1). It was noticed that four bacterial strains of pathogens were detected in the tested samples. These strains are *Staphylococcus*, *Salmonella*, *Listeria*, and *E. coli.* The highest value of total count was recorded for the chickpea tahini paste, while the peanut tahini paste was recorded as high for yeast and mold infection.

### 3.2. Determination of Mycotoxin Contents

**Figure 1:**
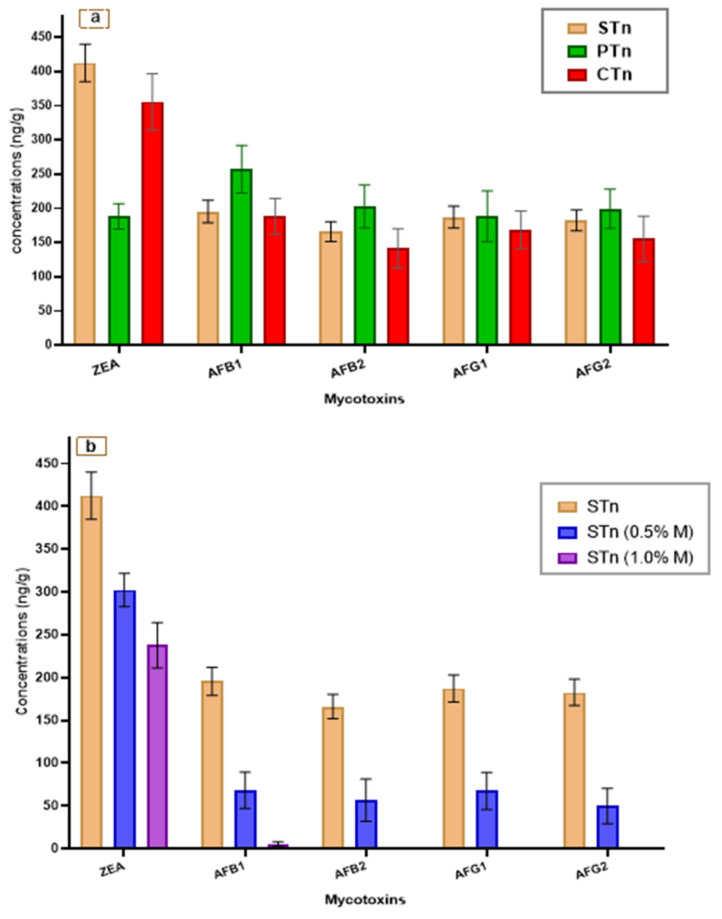
Mycotoxin content of survey samples and fortified samples of tahini. (**a**) Mycotoxin’s natural contamination in common tahini samples; (**b**) mycotoxin content changes after mushroom fortification in the tahini sesame model. AFB1: aflatoxin B_1_; AFB_2_: aflatoxin B_2_; AFG_1_: aflatoxin G_1_; AFG_2_: aflatoxin G_2_. STn: sesame paste tahini; PTn: tahini manufactured with sesame paste containing 10% peanut paste; CTn: tahini manufactured with sesame paste containing 10% chickpea paste.

The mycotoxin contamination of raw tahini samples of the STn, PTn, and CTn was evaluated and is represented in Figure 1a. The results were reflected by high mycotoxin contamination of zearalenone and aflatoxins. The greatest contamination by zearalenone toxin was recorded in samples of sesame tahini, followed by chickpea tahini. In contrast, the peanut paste of tahini was recorded as the lowest one in the ZEA toxin. On the opposite side, the paste of peanut tahini was the greatest contaminated sample of the AFs. However, the paste of sesame tahini recorded considerable content of the ZEA toxin and the AFs, which points to a critical issue.

In contrast, STn, as the model, was evaluated again after its fortification with the lyophilized mushroom powder and stored for 45 days (Figure 1b). The result of fortified samples was shown by the reduction of the contamination level of mycotoxins. Overall, 1% fortification by mushroom samples reflects the best reduction for the ZEA toxin and the AFs. The reduction recorded for the AFs was more effective, where AFB_2_, AFG_1_, and AFG_2_ in 1% fortified samples were not detected. At the application of 0.5% mushroom fortification of sesame tahini samples, the reduction of AFs was considerable but not completely done up to an undetectable level.

### 3.3. Antimicrobial Properties of Agaricus blazei Extract

*Agaricus blazei* extract was evaluated for its antibacterial impact against Gram-positive and Gram-negative strains (Table 2). The results reflect the potency of the extract to inhibit bacterial growth for the tested strains of pathogenic bacteria. It was obvious that there was an enhancement of the antibacterial impact of the extract against pathogen strains of bacteria by the elevation of the applied concentration. Using three concentrations of the extract (10, 20, and 50 µg), the results referred to an increment in the inhibition influence to the tested strains of bacterial pathogens. The highest inhibition zone achieved by the 50 µg extract was recorded for the *Bacillus cereus* strain of Gram-positive pathogens and for the *Salmonella typhi* strain of Gram-negative pathogens. The extract’s minimal inhibitory concentration (MIC) is effective against the Gram-positive and Gram-negative strains. No minimal fungicidal concentration was recorded against toxigenic fungal strains except for the *Fusarium* strain.

### 3.4. Antioxidant Activity and Phenolic Contents

**Figure 2:**
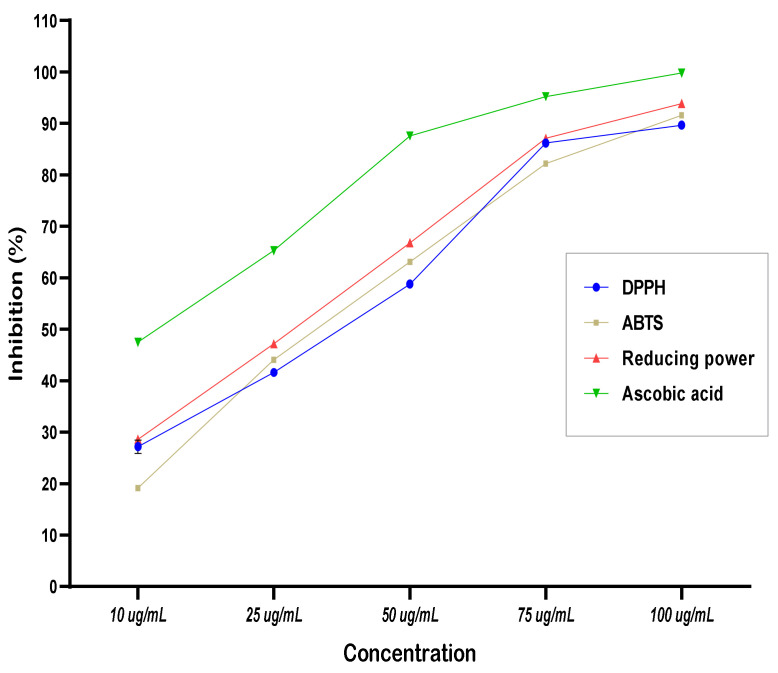
Antioxidant activity of *Agaricus blazei* extract was determined using 3 different assays.

The lyophilized powder of the *Agaricus blazei* extract was evaluated for antioxidant activities, and the results represent a moderate antioxidant potency compared to the standard material of ascorbic acid (Figure 2). The result recorded by the three assays was close, which may explain a part of the extract potency for its activity as an antimicrobial extract. Otherwise, the total phenolic content determined for the extract was recorded as 38.65 ± 3.54 mg GAE/g lyophilized extract. It was noticed that the evaluated extract possesses considerable antioxidant potency. This point could explain the antimicrobial effect recorded in the previous section. Again, the antioxidant activity recorded for the extract may play a critical function in the shelf life of the fortified tahini sample.

### 3.5. Tahini Evaluation

STn was selected as a common tahini type to be applied as a model for further fortification. Chemical composition and rheological parameters were evaluated before and after fortification with the mushroom. Crude fiber and protein content were recorded for the two fortification levels applied (Table 3). Moreover, an increment was recorded for the viscosity of the fortified tahini samples. The color attribute changes (∆E) were not so high for the fortified tahini. The significant point of tahini’s manufacturing quality is the oil separation. The fortification of tahini by *Agaricus blazei* lyophilized powder was very effective in increasing the product stability: the more the fortification ratio, the more the stability parameters, and the lower the oil separation appearance.

### 3.6. Oxidative Stability

Oxidative stability parameters of the fortified tahini products and the control sample were measured after 45 days of normal storage (temperature: 40–42 °C; RH: 86–89%). The peroxide value for the control was 1.05 ± 0.08 mgKOH/g oil. The corresponding values for the fortified samples of F1 tahini oil (0.5%) and F2 tahini oil (1.0%) were 0.97 ± 0.02 and 0.88 ± 0.05 mgKOH/g oil, respectively. The *p*-anisidine value was 1.37 ± 0.05 meq/kg for the control, 1.28 ± 0.02 meq/kg for F1 tahini, and 1.21 ± 0.01 meq/kg for F2 tahini. These results reflect the enhancement of tahini’s oxidative stability due to fortification, particularly with the 1.0% addition of mushroom lyophilized powder.

### 3.7. Bioactivity of Fortified Tahini

**Figure 3:**
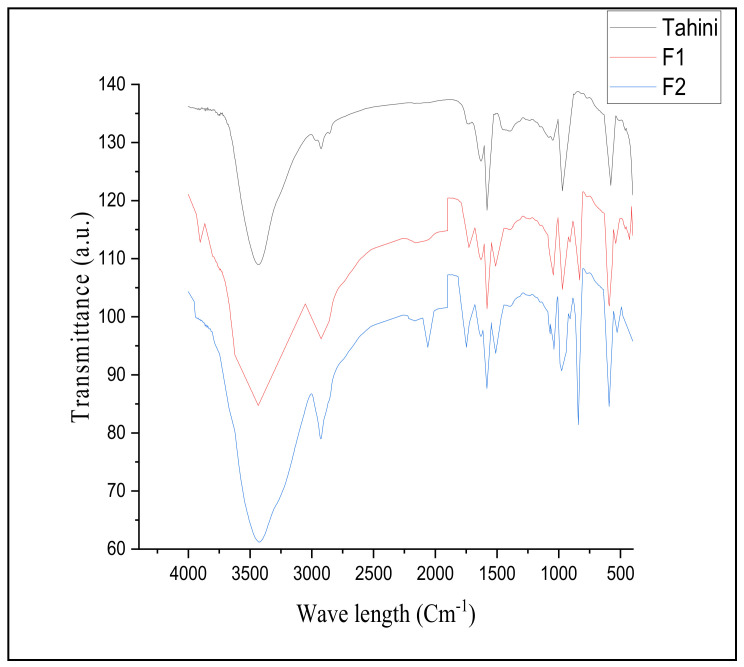
Fourier-transform infrared (FTIR) results for the control and fortified tahini samples. Tahini: the standard sample of tahini manufactured from sesame paste; F1: fortified tahini sample manufactured using 0.5% lyophilized mushroom; F2: fortified tahini sample manufactured using 1.0% lyophilized mushroom.

FTIR is one of the standard techniques for the active group indication of investigated constituents. However, the absorption frequencies of active groups can distinguish the related substances and the contents of the applied components. The functional groups’ content differences are related to their frequencies by comparing the investigated material before and after fortification. The data of the Fourier-transform infrared spectroscopy (FTIR) results in the region between 500 and 1500 cm^−1^ are shown in Figure 3. An increment was traced for tahini fortified with lyophilized mushroom. The activity was more affected by the increment of fortification ratio, and a deeper curve represented the impact. The stretching vibration of a single bond for the hydroxyl group was connected with a strong absorption band of about 3400 cm^−1^ (3410 cm^−1^ or 3370 cm^−1^).

However, the C-H single bond stretching vibration was linked to the weak band at about 2950 cm^−1^ (2910 cm^−1^ or 2960 cm^−1^), which increased by fortification. The stretching of the C-O-C bond vibration was recorded in bands at 1100 cm^−1^. Asymmetrical stretching bands at 1645 cm^−1^ and the weaker symmetric stretching bands near 1410 cm^−1^ were linked to the C=O asymmetric and symmetric stretching. The weak bands around 840 cm^−1^ indicated the presence of the α-linked glycosyl residues, while the stretching of 880 cm^−1^ indicated the presence of the β-linked glycosyl residues.

Notwithstanding, the laccase enzyme activity was recorded as 2050 U/L for the crude centrifuged solution resulting from the soaked powder. However, the ultrafiltered solution recorded has enzyme activity equal to 1680 U/L. Detectable activity and the high capacity of the mushroom enzyme were following a previous study [39]. The result regarding the β-glucan content of lyophilized mushroom powder was 58.1 g/Kg (5.8%). The mushroom content of oligo and polysaccharides was measured as glucose content being 694 µg/g of mushroom lyophilized powder. It is worth referring to the impact of oligo and polysaccharides as bioactive components, particularly for their antimicrobial properties [40,41].

### 3.8. Microbial Content Changes after Fortification

The microbial content of the STn tahini model was reevaluated after the fortification steps. The total bacterial count of fortified tahini was recorded as less than the control sample (Table 4). The most hazardous bacteria (*Salmonella* sp.) were not detected after the fortification and were stored for 30 days under temperature and humidity aseptic conditions. Fortification using the high ratio (1.0%) reflects more microbiological safety characteristics than the lower (0.5%). However, *E. coli* bacteria disappeared at 1.0% fortification, and the decreases recorded for molds and yeast were lower than the antibacterial effect. These results may recommend 1.0% for the fortification step due to the more microbiological quality of the final product after storage.

### 3.9. Sensory Evaluation

**Figure 4:**
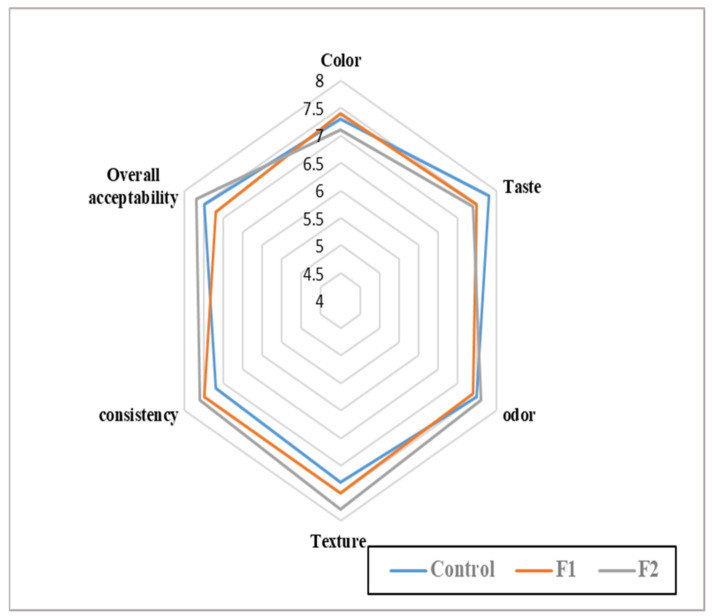
Sensory evaluation of the control and fortified samples of sesame tahini. F1: Sesame tahini contains 0.5% *Agaricus blazei* lyophilized powder; F2: sesame tahini contains 1.0% Agaricus *blazei* lyophilized powder; ND: not detectable; sesame tahini: *tahini* manufactured with just sesame paste.

The sensory evaluation data reflect slight changes in the impression of the panelists against the fortified tahini with mushroom lyophilized powder (Figure 4), while the tahini sample of 1.0% fortification was less acceptable for taste and color than the control. At the same time, it was better for the texture, consistency, and overall acceptability. Obviously, the more microbiological safety parameters recorded for fortified tahini recommended it to be an alternative product because it is safer, more stable, and has low differentiation comparable to the sensory point of view control.

## 4. Discussion

In various ready-to-eat food items, tahini—a popular food item in the Middle East—is a key component. Tahini and its products have been connected to outbreaks of foodborne disease and product recalls across the world [42]. Tahini is renowned for its high fat level (57–65%), low moisture content (1%), 6.4–9.0% carbohydrate content, and 23–27% protein content [11,43]. Tahini is regarded as a ready-to-eat food with an extended shelf life that is kept up to two years at room temperature. It is a frequently used component in numerous ready-to-eat culinary products, including hummus, baba-ghanouj, halva, and other salad dressings [44,45]. The common tahini product known globally is sesame butter [2]; it is mixed in some local areas with peanut butter or chickpea powder for texture enhancement. The present study was first targeted to evaluate commercial tahini’s current situation in local markets. Over the past ten years, tahini and its related products have become more globally popular. For the food industry and regulatory organizations, the microbiological safety of tahini and its derivative products has increased in importance. However, *Salmonella* is a great hazard joined with sesame infection bacteria worldwide [46]. Tahini is thought to be shelf stable due to its low water activity; however, due to its high fat content, it may cause cross-protection against negative environmental factors, which might prolong the survival of pathogens such as *Salmonella* under different storage conditions [47]. The previous studies reported some microbial issues regarding tahini products in markets. The main issue regarding tahini consumers is *Salmonella* contamination occurrence [11,48]. At the same time, other technical issues may affect consumers’ acceptability, such as oil separation and consistency [49].

The current study reported a high microbial count for the collected samples of three tahini types. Several pathogens were recorded, including *Staphylococcus*, *Listeria*, *E. coli*, and *Salmonella* (Table 1). Mold and yeast were also detected in the investigated samples. Moreover, chemical hazards like mycotoxin contamination were also reported [50,51]. *Salmonella* as a bacterial infection may contaminate the paste of tahini due to the insufficient roasting degree of sesame seeds [52] and contamination may happen after heat treatment as a result of unsanitary conditions during production and tahini products packing [48].

Collected samples of tahini were examined for their content of aflatoxins (AFB1, AFB2, AFG1, and AFG2) and zearalenone (ZEA). The four types of aflatoxins existed in all investigated samples, which referred to an actual chemical hazard hidden in tahini products. The results reflected a high content of the ZEA for the two tahini types of STn and CTn (Figure 1a). Meanwhile, the AFs exhibited high content in the PTn type of tahini; this may be connected to more aflatoxins contamination in peanuts [13,19].

Aflatoxins are great hazard chemicals joined to fungi-producing toxins. Concerning the European Commission and Egyptian Standards, a maximum permitted limit for aflatoxins in oilseeds, including sesame products, was defined for AF summation at 4 μg/kg and 2 μg/kg for the AFB_1_ individually [51]. The results reflected in Figure 1b show the limitation of aflatoxin concentrations to meet the regulation by the fortification ratio of (0.5%), while 1% of mushroom fortification in tahini leads to undetected levels of aflatoxins in the product. Several investigations have reported mushroom species as a source of bioactive components and active enzymes [28,40,53]. The extract of mushrooms exhibited a considerable antibacterial impact against several investigated strains of Gram-positive and Gram-negative bacteria (Table 2). The effect of the extract was not high against toxigenic strains of fungi in the microbial growth media. The impact extract of the mushroom as a high antimicrobial agent could be explained due to the high antioxidant activity determined against the standard of ascorbic acid (Figure 2). In the same situation, a large content of phenolic compounds (38.65 ± 3.54 mg GAE/g) was recorded for the lyophilized mushroom extract.

Depending on the preliminary experiments in laboratory trials evaluated by the specialist panelists, two ratios of mushroom fortification were chosen to apply in tahini (0.5% and 1.0%). One type of tahini (STn) was also chosen as a model for the application and evaluation in the next steps. The evaluation of fortified samples against the control reflects changes in the proximate analysis, particularly increasing protein and fiber content. Little changes were recorded in the color attributes of fortified tahini, and elevation in viscosity was also recorded. The relevance of this process is reflected in the color characteristics, water activity, and viscosity analyses that were determined to quantify the changes that occurred to tahini after fortification. The product color change rate was the lowest after applying lyophilized mushroom powder (Table 3). The water activity of the tahini samples was reduced by fortification, indicating that the fortified products had less microbiological activity over the storage time. Tahini stability and its little oil separation are preferable properties requested in the product. In this regard, less oil-separated tahini is a quality characteristic that needs to be achieved in tahini. An amelioration reflected the fortification process by the two ratios in the emulsion efficiency ratio compared to the control sample. The impact of the panelists’ evaluation test was recorded by a few changes in the fortified tahini with acceptable properties.

In the same way, the oxidative stability of fortified tahini samples was reflected by amelioration achieved in the final product after 45 days of storage time. These results could indicate a shelf-life extension achieved by the fortification step for the tahini. The values of oxidative stability referred to 1.0% fortification as a more effective ratio. The tahini content of bioactive material that occurred due to the fortification was also evaluated utilizing the FTIR technique (Figure 3). Enrichment for the region of 500–1500 cm^−1^ stretching was recorded by the fortification step, where this region was linked to bioactive oligo and polysaccharides. Again, an increment was recorded for the region of about 3400 cm^−1^ related to the hydroxyl group activity. The increasing bioactive group was also recorded for amid and amino groups. Commonly, absorption frequencies of the area range between 500 and 1500 cm^−1^, which is differentiated regarding the content of NO_2_ stretch, C-OH stretch, C-O-C stretch, and C=C stretch. This region is usually a fingerprint to clarify the treatment impact [54]. The bands regarding 840 cm^−1^ stretching referred to the α-linked existence of glycosyl residues, and the 880 cm^−1^ stretching region points out the β-linked presence of the glycosyl residues. These components are in agreement with the curve explained previously [53].

As a model, the tahini sample of the initial samples collected was utilized to evaluate the impact of fortification against the total bacterial count and the mycotoxin content. The results expressed the ameliorative effect of the mushroom lyophilized powder application for decreasing the microbial content (Table 4). The most hazardous strains of *Salmonella* sp., *Staphylococcus* sp., and *Listeria* were not detectable by a 1.0% fortification ratio. The impact of the fortification process could be explained on the basis of the distinguished content of antioxidant potency (Figure 2). Bioactive content of oligo and polysaccharides (Figure 3) recorded for the tested tahini sample after fortification also could possess a synergistic effect with antioxidant activity and phenolic content impacts [32]. At the same fortification ratio, the degradation in mycotoxin content was also recorded as having a considerable effect (Figure 1). This impact concerns the bioactive content of lyophilized powder applied, such as β-glucan [26], oligo and polysaccharides [55], laccase activity [56,57], and other bioactive peptides indicated by the FTIR curves.

## 5. Conclusions

A survey for the common tahini samples of STn, PTn, and CTn reflected a high incidence of microbial pathogen strains and mycotoxin content contamination. *Agaricus blazei* is an edible mushroom fungal plant with medicinal impact and high bioactive content. *Agaricus* was lyophilized to keep the enzyme activity and the bioactive content efficient. Lyophilized powder was extracted and evaluated for antioxidant potency, phenolic content, bioactive polysaccharides, enzyme activity, and antimicrobial activity. Two ratios of fortification (0.5% and 1.0%) utilizing the lyophilized powder were applied in sesame butter tahini to overcome the contamination issues and enhance the quality characterization. The evaluation of fortified tahini reflects acceptable characteristics of color attributes, chemical composition, viscosity parameters, emulsion stability, and oxidative stability. The application of fortification ratios was shown by the ameliorative impact on pathogenic contamination and mycotoxin degradation. A common hazard in the exported tahini, *Salmonella*, was not detected in the 1.0% fortified sample. Oxidative stability, microbial evaluation, and mycotoxin content examined after the fortification process recommended 1.0% fortification as a shelf-life extension process, quality enhancer, and microbial safer and adequate treatment in tahini products.

## Figures and Tables

**Table 1:** Average contamination levels in bulk tahini samples handled commonly in markets (logcfu/g).

	Sesame Tahini	Peanut Tahini	Chickpea Tahini
**TBC**	3.34–5.72	2.41–4.49	3.85–5.57
***Staphylococcus* sp. **	0.47–1.69	0–0.95	0.0–0.84
***Salmonella* sp. **	0.60–1.04	0–0.47	0.0–0.69
***Listeria* sp. **	0.47–0.90	0.0	0.0–0.30
***E. coli***	2.5–2.69	2.25–2.32	2.04–2.27
***Yeasts***	1.81–1.93	1.32–1.59	0.77–1.14
***Molds***	1.61–1.94	1.94–2.13	1.80–2.02

The data expressed in the table are calculated as the average number of microorganisms among the collected samples (*n* = 20). TBC: total bacterial count; sesame tahini: tahini manufactured with just sesame paste; peanut tahini: tahini manufactured with sesame paste containing 10% peanut paste; chickpea tahini: tahini manufactured with sesame paste containing 10% chickpea paste.

**Table 2:** Antibacterial and antifungal properties of *Agaricus blazei* extract.

	**IZD (mm)**			**MIC**	**MBC**	**MFC**
	10 µg	20 µg	50 µg	(µg/L)	(µg/L)	(µg/L)
**Gram-positive Bacteria**						
***Staphylococcus aureus ATCC 13565***	11.21 ± 1.05	18.1 ± 1.14	26.1 ±0.74	250	350	--
***Bacillus cereus EMCC 1080***	11.6 ± 0.54	16.3 ± 1.08	28.3 ± 1.31	250	350	--
***Enterococcus faecalis ATCC 51299***	12.4 ± 0.88	18.5 ± 1.24	26.2 ± 1.02	200	350	--
**Gram-negative Bacteria**						
***Escherichia coli ATCC 51659***	8.25 ± 2.05	15.66 ± 1.54	22.18 ± 1.64	300	450	--
***Salmonella typhi ATCC 15566***	6.88 ± 1.76	16.41 ± 2.33	23.05 ± 1.81	300	450	--
***Listeria monocytogenes ATCC 7644***	8.57 ± 2.08	14.97 ± 1.87	21.88 ± 2.25	300	450	--
**Fungi**						
***A. flavus*** ***ITEM* 698**	ND	1.58 ± 0.14	4.21 ± 0.24	--	--	ND
***A. niger ITEM 9568***	ND	1.94 ± 0.22	5.77 ± 0.41	--	--	ND
***A. ochraceus ITEM 7043***	ND	2.82 ± 0.55	4.86 ± 0.88	--	--	ND
***P. chrysogenum ATCC 10106***	ND	3.27 ± 1.47	5.06 ± 1.05	--	--	ND
***F. culmorum KF 846***	2.54 ± 0.46	6.14 ± 1.34	8.74 ± 1.24	--	--	850

The data were expressed as means ± SD (where *n* = 3); ND: represents the not detected compounds. The impact was measured for 3 concentrations (10, 20, and 50 µg lyophilized extract/L media). MIC: minimal inhibitory concentration; MBC: minimal bactericidal concentration; MFC: minimal fungicidal concentration. IZD: inhibition zone diameter measured in millimeters.

**Table 3:** Chemical composition and rheological changes of sesame tahini after *Agaricus blazei* fortification.

**Tahini Chemical Composition**							
	Moisture content	a_W_	Crude fat	Crude fiber	Protein	Total sugars	Total ash
STn	2.05 ± 0.22	0.192 ± 0.002	55.22 ± 1.54	3.09 ± 0.05	22.91 ± 0.64	8.52 ± 0.88	2.88 ± 0.08
STn (0.5%)	1.67 ± 0.14	0.171 ± 0.005	52.12 ± 0.87	4.87 ± 0.08	26.21 ± 0.89	6.12 ± 1.05	3.05 ± 0.11
STn (1%)	1.16 ± 0.18	0.154 ± 0.001	48.87 ± 0.64	6.27 ± 0.06	29.34 ± 0.96	5.81 ± 1.12	4.02 ± 0.05
**Viscosity, Color Parameters, and Stability**							
	L*	a*	b*	∆E	Viscosity (Cp)	% EE	
STn	55.37 ± 0.81	5.61 ± 0.14	12.23 ± 0.21	--	2577 ± 2.5	84.9 ± 1.24	
STn (0.5%)	57.05 ± 0.37	3.27 ± 0.11	12.71 ± 0.34	8.53	2990 ± 4.1	91.05 ± 1.66	
STn (1.0%)	58.17 ± 0.24	2.81 ± 0.06	13.11 ± 0.37	16.45	3880 ± 3.6	95.41 ± 0.56	

The data were expressed as means ± SD (where *n* = 3); STn: sesame tahini; STn (0.5%): sesame tahini contains 0.5% *Agaricus blazei* lyophilized powder; STn (1.0%): sesame tahini contains 1.0% *Agaricus blazei* lyophilized powder; Cp: centipoise; a_W:_ water activity; EE: emulsion efficiency of tahini sample.

**Table 4:** Microbiological changes in the tahini model after fortification by *Agaricus blazei* (logcfu/g).

	Sesame Tahini	F1	F2
**TBC**	4.85	2.04	1.94
***Staphylococcus* sp. **	1.63	ND	ND
***Salmonella* sp. **	1.11	ND	ND
***Listeria* sp. **	0.95	ND	ND
***E. coli***	2.75	0.69	ND
***Yeasts***	1.95	1.38	0.61
***Molds***	1.98	1.78	1.44

The data expressed in the table is calculated as the average number of microorganisms among the collected samples (*n* = 20). TBC: total bacterial count; F1: sesame tahini contains 0.5% *Agaricus blazei* lyophilized powder; F2: sesame tahini contains 1.0% *Agaricus blazei* lyophilized powder; ND: not detectable. Sesame tahini: tahini manufactured with just sesame paste.

## Data Availability

All data regarding this work were represented inside the manuscript.
